# Dynamic Interaction of Enterovirus 71 and Dendritic Cells in Infected Neonatal Rhesus Macaques

**DOI:** 10.3389/fcimb.2017.00171

**Published:** 2017-05-10

**Authors:** Ting Zhao, Zhixiao Zhang, Ying Zhang, Min Feng, Shengtao Fan, Lichun Wang, Longding Liu, Xi Wang, Qinglin Wang, Xiaolong Zhang, Jingjing Wang, Yun Liao, Zhanlong He, Shuaiyao Lu, Huai Yang, Qihan Li

**Affiliations:** Yunnan Key Laboratory of Vaccine Research and Development on Severe Infectious Diseases, Institute of Medical Biology, Chinese Academy of Medical Sciences and Peking Union Medical CollegeKunming, China

**Keywords:** Enterovirus 71 (EV71), dendritic cells, neonatal rhesus macaques, infection, respiratory tract

## Abstract

Enterovirus 71 (EV71) is one of the main pathogens responsible for hand, foot, and mouth disease (HFMD). Infection with EV71 can lead to severe clinical disease via extensive infections of either the respiratory or alimentary tracts in children. Based on the previous pathological study of EV71 infections in neonatal rhesus macaques, our work using this animal model and an EV71 chimera that expresses enhanced green fluorescent protein (EGFP-EV71) primarily explored where EV71 localizes and proliferates, and the subsequent initiation of the pathological process. The chimeric EGFP-EV71 we constructed was similar to the wild-type EV71 (WT-EV71) virus in its biological characteristics. Similar clinical manifestations and histo-pathologic features were equally displayed in neonatal rhesus macaques infected with either WT-EV71 or EGFP-EV71 via the respiratory route. Fluorescent signal tracing in tissues from the animals infected with EGFP-EV71 showed that EV71 proliferated primarily in the respiratory tract epithelium and the associated lymphoid tissues. Immunofluorescence and flow cytometry analyses revealed that EV71 was able to enter a pre-conventional dendritic cell (DC) population at the infection sites. The viremia identified in the macaques infected by WT-EV71 or EGFP-EV71 was present even in the artificial presence of a specific antibody against the virus. Our results suggest that EV71 primarily proliferates in the respiratory tract epithelium followed by subsequent entry into a pre-cDC population of DCs. These cells are then hijacked by the virus and they can potentially transmit the virus from local sites to other organs through the blood circulation during the infection process. Our results suggest that the EV71 infection process in this DC population does not interfere with the induction of an independent immune response against the EV71 infection in the neonatal macaques.

## Introduction

Enterovirus 71 (EV71), a member of the *Enterovirus* genus with a small viral RNA structure, is widely recognized as one of the major pathogens responsible for the large outbreaks of hand, foot, and mouth disease (HFMD) in children in the Asian-Pacific region (McMinn, 2002). EV71 not only leads to HFMD, as displayed by vesicular lesions, but also sometimes causes severe neurological injury and even death, as has been described in a clinical study (Chang et al., 1999; Ooi et al., 2010; Solomon et al., 2010). The pathological progress of the disease, whose mechanism remains largely unknown, is frequently accompanied by a transient elevation of several pro-inflammatory cytokines in the peripheral blood and cerebrospinal fluid, in the absence of an abnormal immune response (Lin et al., 2003; Zhang et al., 2011; Griffiths et al., 2012; Xu et al., 2013; Chen et al., 2014). Although, the recently licensed inactivated EV71 vaccine can prevent this viral infection and its related clinical disease (Li et al., 2014), further investigation of the pathogenesis caused by EV71 and its relationship with the immune system will lead to the better control of this epidemic disease until the vaccine is more widely implemented.

A previous study by He et al. using viable tissues from autopsy cases suggested that the tonsillar crypt epithelium was an important extra-central nervous system site for viral replication in EV71 encephalomyelitis (He et al., 2014). This suggests that lymphokinesis might provide a pathway for viral infection. Recent work has also confirmed that EV71 can infect human dendritic cells (DCs), and that when infected these cells can stimulate and activate host T cell responses (Lin et al., 2009). Furthermore, the data from our previous study indicated that the virus was capable of infecting CD14^+^ cells (Wang et al., 2013), which are immature DC types (Rossi and Young, 2005). Because infection with EV71 can induce a distinct specific immune response (Liu et al., 2013), the above data might support the hypothesis that EV71 infection of DCs is correlated with the interaction between the virus and immune system, thereby leading to up-regulated expression of cytokines such as IL6 and TNFα (Liu et al., 2013). If this is the case, evaluating how the immunity induced by the vaccine influences the interaction between the virus and the immune system is very important. The first step for investigating this is to focus on the dynamic interaction between the virus and dendritic cells during the infection. Based on such analyses, we previously established a neonatal rhesus macaque model for EV71 infection, in which EV71 was capable of infecting the macaques through the respiratory tract. In this model, the typical clinical pathological process resulting in vesicular lesions in oral mucosa and limb skin, fever and viremia was observed (Dong et al., 2010; Liu et al., 2011).

In the present study, we investigated the dynamic interaction between DCs and EV71 upon viral entry into macaques by using an EV71 chimera (called EGFP-EV71) that expresses enhanced green fluorescent protein (EGFP). The results obtained show that the epithelial DCs are involved in early events of EV71 infection. DCs are infected by virus in the mucosal tissues of the respiratory tract after which they migrate to the associated lymph nodes. This process triggers the DCs to act as carriers for the virus from the lymphatic system to the peripheral blood system, after which the virus is subsequently transferred to its target organs.

## Methods

### Viruses and cells

The FY-23 virus used in this study was isolated from a patient from the HFMD outbreak in Fuyang, Anhui Province, China in 2008 (GenBank accession number: EU812515; Ma et al., 2009). Vero cells (ATCC, Manassas, VA, USA), cultured in minimum essential medium (MEM; Invitrogen, Carlsbad, CA, USA) containing 10% newborn calf serum (NBCS) (HyClone, Logan, UT, USA), were subjected to viral proliferation, titration, and other analyses.

### Construction of complete EV71 genomic clones

The RNA genome of the FY-23 virus was extracted using the MiniBEST Viral RNA/DNA Extraction Kit Version 3.0 (Takara Shuzo, Shiga, Japan) according to the manufacturer's instructions. Complementary DNA was prepared using reverse transcriptase (PrimeScript II RTase, Takara) and the EV717409R (5′-TTTTTTTTTTTTTTTTTTTTTTTTTGGCTATTCTGGTTA-3′) primer, which is based on the consensus 3′-sequence of the FY-23 virus RNA-end sequence. The first (1–3,711 nt) and the second fragments (3,687–7,409 nt, including a 26-base poly A tail) of EV71 were PCR-amplified using complementary DNA, primer pairs, and DNA polymerase (PrimeSTAR GXL, Takara). The first and second fragment primer pairs were complementary to parts of the 1–3,711 nt region and the 3,687–7,409 nt region of the FY-23 RNA genome, respectively. The primers for the first fragment were 5′-ACGAAGCTTTTAAAACAGCCTGTGGGTTGCAC-3′ and 5′-CCAGTCGACGCTATGCCGACGACGGCA-3′ (the underlined nucleotide base was an A to C synonymous mutation, which introduced a *Sal* I cloning site). The primers for the second fragment were 5′-GCGTCGTCGGCATAGTGTCGACTGGTGGC-3′ and 5′-TGCGAGCTCTTTTTTTTTTTTTTTTTTTTTTTTTTGATTCGTTGTA-3′. The first fragment was cloned into the unique *Hin*d III and *Sal* I cloning sites of the pSP64 vector (Promega, Madison, Wisconsin, USA), and the second fragment was sub-cloned into the unique *Sal* I and *Sac* I cloning sites of the pSP64 vector, thereby constructing clones containing the complete EV71 genome.

### Construction of the EGFP-labeled EV71 chimera (EGFP-EV71)

**Figure 1:**
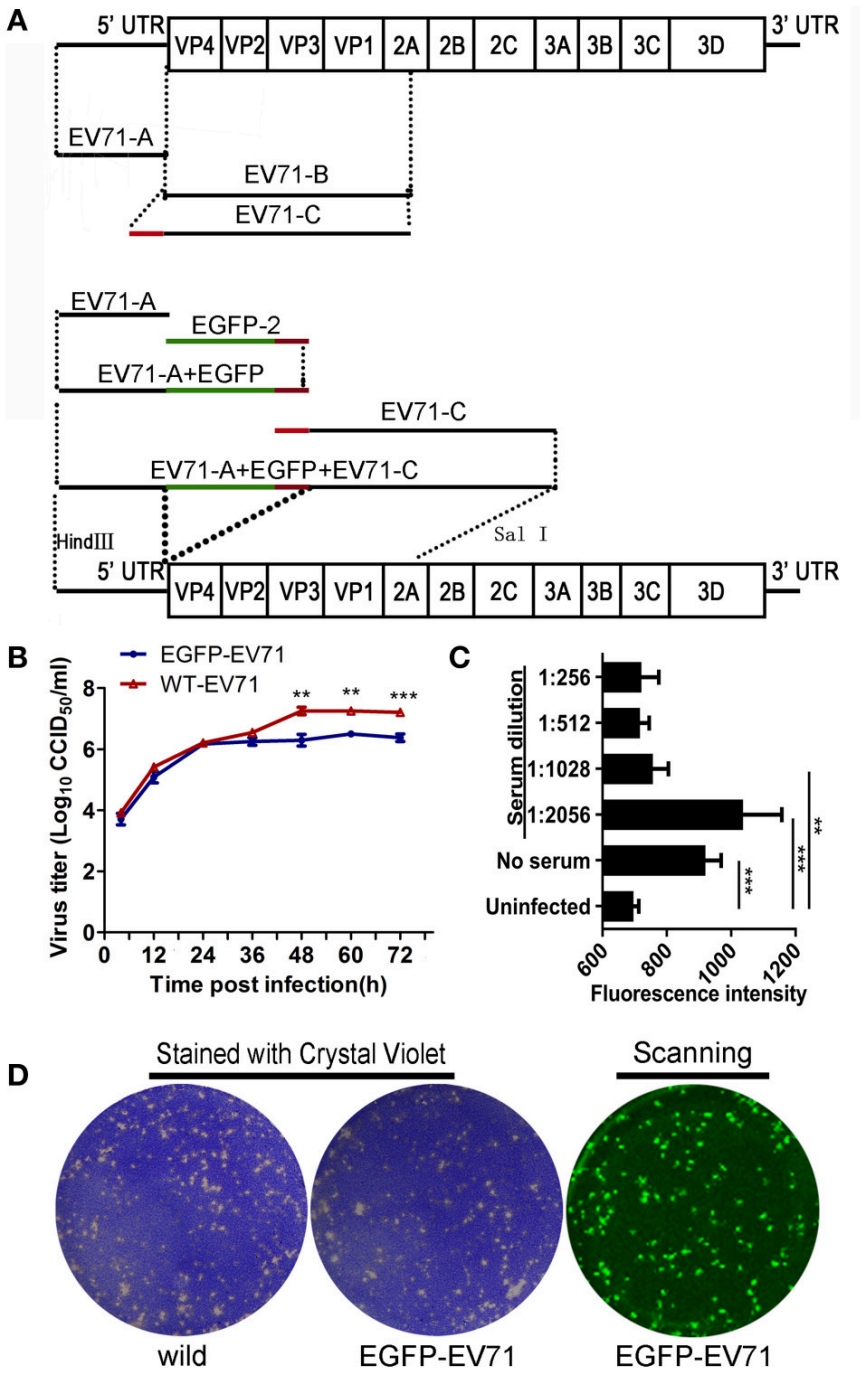
**Construction of the fluorescently labeled EGFP-EV71 virus and its phenotype analysis. (A)** Construction of the fluorescently labeled EGFP-EV71 virus. The green line represents the EGFP fragment and the red line represents the fragment encoding the AITTLGS peptide, the latter of which is capable of being recognized as a cleavage site by the viral-encoded 2A enzyme. **(B)** EGFP-EV71 and WT-EV71 growth curves in Vero cells. The data are presented as the mean ± SD of three independent replicates. **(C)** The level of EGFP-EV71 neutralization by an anti-EV71 antibody. The fluorescence intensities of EGFP were detected with exciting light: 485/20 nm and emission light: 528/20 nm. The significance of each difference was determined using an unpaired Student's *t*-test, two-tailed; ^**^p < 0.01, and ^***^p < 0.001. **(D)** The plaques formed by EGFP-EV71 and WT-EV71 in Vero cells were stained using crystal violet and scanned to locate the fluorescence as described in the Materials and Methods Section.

Figure 1A shows the overall scheme used for cloning the full-length EV71 cDNA. By using the full-length EV71 cDNA clone as a template, two cDNA fragments (EV71-A, 1–754 and EV71-B, 745–3,711) were PCR-amplified with high fidelity DNA polymerase (PrimeSTAR GXL) and two pairs of primers. The primer sequences are listed in Table 1. Nucleotides encoding EGFP were amplified from the pEGFP-N2 vector (Clontech, San Francisco, CA, USA) using the EGFP-1F and EGFP-1R (Table 1) primers and DNA polymerase (PrimeSTAR GXL). The primer sequences marked with underscore represent the nucleotide-encoding peptide (AITTLGS) cleavage recognition site recognized by the viral-encoded 2A enzyme. The final sequence was inserted between EGFP and EV71-B. EV71-A, EV71-B, and EGFP-1 fragments were purified using a TaKaRa MiniBEST Agarose Gel DNA Extraction Kit (Takara). To generate the overlapping sequences required for connecting the fragments we used an overlap extension PCR technique (Heckman and Pease, 2007). The EGFP-2 fragment was PCR-amplified from the EGFP-1 template using EGFP-2F, and EGFP-2R as primers, and the EV71-C fragment was PCR-amplified using EV71-B as the template and EV71-*Hin*dIII-1F and EV71-X-754R as the primers. First, the overlap extension PCR technique was used with EV71-A and EGFP-2 as templates, thereby producing the EV71-A+EGFP fragment. Second, by using EV71-A+EGFP and EV71-C as templates, the EV71-A+EGFP+EV71-C fragment was PCR-amplified. Finally, the EV71-A+EGFP+EV71-B fragment was cloned into the unique *Hin*d III and *Sal* I cloning sites of pSP64- EV71, resulting in the full-length cDNA clone, pSP64-EV71-EGFP. A recombinant plasmid carrying EGFP was linearized and used as a template for *in vitro* transcription, which was performed using the Ribo-MAX Large-Scale Production System (Promega). Complete RNA transcripts were transfected into Vero cells (cultured in 6-well plates and transfected at 5 μg/well) using Lipofectamine 2000 (Invitrogen). When typical cytopathic effects (CPEs) and green fluorescence were observed, EGFP-EV71 was harvested using low-speed centrifugation (4°C, 10 min, 2,000 × g), and the supernatants were stored. The DNA sequences were confirmed for all the plasmids described.

**Table 1:** **Primer sequences**.

**Primer**	**Sequence(5′ to 3′)**	**Fragment**
EV71-HindIII-1F	TACAAGCTTTTAAAACAGCCTGTGGGTTGCAC	EV71-A
EV71-754R	TTGCTCACCGAACCCATGTTTAGCTGTGT	
EGFP-1F	CATGGGTTCGGTGAGCAAGGGCGAG	EGFP-1
EGFP-1R	AGGGTAGTAATGGCCTTGTACAGCTCGTCCAT	
EGFP-2F	TAACACAGCTAAACATGGGTTCGGTGAGCAAG	EGFP-2
EGFP-2R	CACTTGTGAGCCAAGGGTAGTAATGGCCTT	
EV71-745F	CCCTTGGCTCACAAGTGTCTACACAGCG	EV71-B
EV71-SalI-3711R	CCAGTCGACACTATGCCGACGACGC	
EV71-HindIII-1F	TAC(AAGCTT)TTAAAACAGCCTGTGGGTTGCAC	EV71-C
EV71-X-754R	CAAGGCCATTACTACCCTTGGCTCACAAGTG	

*The primer sequences marked with underlined Letters represent the nucleotide-encoding peptide (AITTLGS) cleavage recognition site recognized by the viral-encoded 2A enzyme*.

### Viral growth analysis in cells

Growth curves were generated to compare the replication characteristics of EGFP-EV71 with that of the wild-type EV71 (WT-EV71). Confluent monolayers of Vero cells were inoculated with diluted virus at a multiplicity of infection (MOI) of 1 in a 25-cm^2^ flask. After 1-h incubation at 37°C, the viral suspension was removed and replaced with 10 ml of MEM containing 2% NBCS. After 4, 12, 24, 36, 48, 60, or 72 h, the viral cultures were frozen and thawed for virus collection, and the virus titer was determined using a microtitration assay. Briefly, the virus samples were diluted 10-fold sequentially and used to inoculate monolayers of Vero cells in a 96-well plate at 50 μl per well, and each dilution was tested in eight wells. After inoculation, the cells were incubated for 72-h at 37°C, and the viral titer generated was determined based on the extent of the CPE in the inoculated cells. The cell culture infectious dose 50% (CCID_50_) was calculated based on the Reed–Muench method (Pizzi, 1950).

### Virus neutralization testing

We used an antiviral serum collected from a convalescent rabbit previously infected with WT-EV71 to test whether EGFP-EV71 could be neutralized. EV71 virus samples were each diluted to a MOI of 0.01, and the antiviral serum was diluted two-fold, serially. Each well in the 96-well plates was filled with 50 μl of diluted EV71 and 50 μl of diluted antiviral serum. The plates were incubated at 37°C in a CO_2_ incubator, after which the mixtures were added to the cultured Vero cells, and each serum dilution was tested in eight wells. The CPEs were assessed at 72 h post-infection (hpi). At 72 hpi, the plate was read for the intensity of EGFP fluorescence using automatic microplate reader (Synergy 4; Biotek, Winooski, VT, USA), with the filter set (excitation: 485/20 nm, emission: 528/20 nm). Complete neutralization of EGFP-EV71 was defined as not differing significantly from that of the uninfected Vero cells.

### Plaque formation assay

A plaque assay was performed in 6-well plates, each containing a monolayer of 90% confluent Vero cells. The cells were infected with 10-fold dilutions of virus in a total volume of 0.5 ml MEM. After incubating the cells for 1 h at 37°C, the virus suspension was removed and each well was covered with an agar overlay containing 1× MEM, 3% NBCS, and 1% agar. The plates were incubated for 7 days at 37°C in a CO_2_ incubator. At the end of the incubation period, the cells were fixed with formaldehyde and stained with 0.1% crystal violet using a previously described standard protocol (Holland and McLaren, 1959). For the EGFP-EV71 plaque assay, the plates were scanned using a variable mode imager (Typhoon 9410; GE Healthcare, Piscataway, NJ, USA) to identify EGFP-expressing plaques.

### Ethics statement

The animal experiment procedures used in this study were approved by the Experimental Animal Administration and Ethics Committee of the Institute of Medical Biology, Chinese Academy of Medical Sciences & Peking Union Medical College (Approval number: [2014]26). The experiments were performed according to the “Guide for the Care and Use of Laboratory Animals” (National Research Council (US). Committee for the Update of the Guide for the Care and Use of Laboratory Animals., I.F.L.a.R.U.S., 2011) and “The Guidance to Experimental Animal Welfare and Ethical Treatment” (China, 2006).

The animals were reared in cages with sizes exceeding the stipulated minimum (BSL-2 conditions) and these cages allowed for visual, olfactory and auditory interactions with other monkeys. Installed in each room was a temperature control valve guaranteed to maintain the room temperature at ~25°C, and sufficient fresh air and natural light was supplied. Food, water, and fruit were readily available. Appropriate treats and vitamins were provided. The animals were given access to environmental enrichment (such as approved toys) to promote their psychological well-being. All animals were fully under the care of veterinarians at the Institute of Medical Biology (IMB), Chinese Academy of Medicine Science (CAMS). Animals were euthanized using ketamine (Phoenix Pharmaceuticals, St. Joseph, Mo.) as the anesthetic (10 mg/kg of body weight).

### EGFP-EV71 virus infection of neonatal rhesus macaques

A total of 20 healthy neonatal rhesus macaques (2–3 months of age; IMB, CAMS, China) were divided into three groups: the EGFP-EV71-infected group, the WT-EV71-infected group, and the negative control group (phosphate-buffered saline, PBS).

In the EGFP-EV71-infected group, 12 neonatal macaques were challenged with EGFP-EV71 at a dose of 10^4.5^ CCID_50_/macaque. Of these rhesus macaques, six were challenged via the respiratory tract using a virus solution spray, and the other six were challenged via the alimentary tract by feeding the animals with 1 ml (10^4.5^ CCID_50_/ml) of virus solution per macaques. Blood and stool samples were collected daily, and the viral loads were measured. Lymphocytes were isolated using flow cytometry. Six macaques (three infected via the respiratory tract and three infected via the alimentary tract) were euthanized at 3 days post-inoculation (dpi), as well as at 6 dpi. Their organs were temporarily stored in formaldehyde and liquid nitrogen for the subsequent experiments.

In the WT-EV71-infected group, six macaques were challenged with WT-EV71 (dose, 10^4.5^ CCID_50_/macaque). These macaques were challenged with the virus following the same procedures described above for the EGFP-EV71-infected group (three macaques were infected via the respiratory tract and three macaques were infected via the alimentary tract). Two macaques (one infected via the respiratory tract and one infected via the alimentary tract) were euthanized at 3 dpi, as well as at 6 dpi.

The control macaques were each given an equal amount of PBS. One group of macaques was euthanized at 3 dpi, as well as at 6 dpi.

### Frozen tissue sectioning and imaging analysis

The tissues stored in liquid nitrogen were embedded in JUNG tissue freezing medium (Leica, Wetzlar, Germany) and sectioned using a cryostat microtome (CM1950; Leica) according to the manufacturer's protocols. Frozen sections were observed by fluorescence microscopy (DMI6000B; Leica), and the fluorescence intensity was subjected to gray-scale analysis (Leica MM AF Series).

### Fluorescence confocal microscopy and immunofluorescence

The frozen tissue sections were fixed using acetone, and then blocked using 4% bovine serum albumin (BSA) in PBS. Primary antibodies (Supplemental Table 1) were incubated with the sections at 4°C overnight. After blocking, the sections were incubated with a second fluorescently-labeled antibody (Supplemental Table 1) and then observed under a confocal microscope (TCS SP8; Leica).

### Virus load assay

RNA was extracted from blood (200 μl) or tissue samples (100 mg) using the Qiagen RNeasy Mini Kit according to the manufacturer's protocol (Qiagen, Hilden, Germany). Viral loads were detected using the One-step PrimeScript RT-PCR Kit (Takara) according to the manufacturer's protocol, and the 7,500 Fast Real-time RT-PCR System (Applied Biosystems, Foster City, CA, USA). The primer pair and probes used in this protocol were designed to detect the VP1 region (3,172–4,062 nt) of the EGFP-EV71 genome (forward primer: 5′-CACCTGCGAGTGCTTATCAA-3′, forward probe: 5′-FAM-CCACATTCGGAGAACACAAACAGGAGA-BHQ1-3′, reverse primer: 5′-CCTGACGTGCTTCATTCTCA-3′). The real-time PCR amplification conditions used were as follows: 5 min at 42°C and 10 s at 95°C, followed by 40 cycles of 95°C for 5 s and 60°C for 34 s. Viral copies were quantified spectrophotometrically based on the amount of *in vitro*-synthesized complete RNA, and the result was expressed as the relative copy number according to the following equation [(μg of RNA/μl)/(molecular weight)] × Avogadro's number = viral copy number/μl.

### Isolation of peripheral blood mononuclear cells (PBMCs) and single-cell suspension preparations

PBMCs, isolated from rhesus macaque blood samples using Ficollpaque plus (GE) according to the manufacturer's instructions, were resuspended in chilled PBS for use in the flow cytometry assays. When working with tissues, the tissues were gently homogenized using a mortar and pestle, and then washed with 1 ml of PBS through a cell strainer (70-μm BD Bioscience, Heidelberg, Germany) mounted on top of a 50-ml tube (BD Bioscience). The tubes covered with the strainers were centrifuged at 300 × g for 5 min. The resulting cell pellets were each resuspended in PBS to produce individual single-cell suspensions. The resulting number of cells within each tube was analyzed by the direct counting method.

### DC isolation and cultivation

PBMCs were isolated from blood samples using Ficollpaque plus (GE) according to the manufacturer's instructions, and the DCs were isolated from the PBMCs using a blood dendritic cell isolation kit II (MACS, Miltenyi Biotech, Bergisch-Gladbach, Germany) according to the manufacturer's instructions. DCs were resuspended in RPMI-1640 medium supplemented with 10% fetal bovine serum (FBS, Hyclone) and seeded at 5 × 10^6^ cells/ml in 24-well plates.

### Infection of DCs

DCs were incubated with EGFP-EV71 (MOI = 1) for 1 h at 37°C. The cells were then washed to remove the cell-free viruses, resuspended in RPMI-1640 medium supplemented with 8% FBS, and then incubated at 37°C under 5% CO_2_. At the given time points (12, 24, 36, 48, and 60 h) post-inoculation, 200 μl of culture supernatant was collected for virus titration and cytokine measurement. After 24–36 hpi, the inoculated DCs were collected for detection of matured DCs. At 48 hpi, the DCs were collected for flow cytometric analysis.

### Cytokine measurements

IFN-α, IFN-β, IFN-γ, TNF-α, IL-6, and IL-8 in the supernatants mentioned above were measured with ELISA kits (Cusabio Biotech, Wuhan, China) according to the manufacturer's instructions.

### Flow cytometry

Cells were washed and blocked in staining buffer (PBS with 0.3% BSA and 0.1% sodium azide) containing anti-CD16 and anti-CD32 (BD Biosciences, San Jose, CA, USA) for 10 min at 4°C, and then stained with fluorophore-conjugated secondary antibodies. After washing the cells twice with staining buffer, flow cytometry data were acquired using a FACSCanto II flow cytometer (BD Biosciences) and analyzed using FlowJo software (Treestar, Ashland, OR, USA).

Cells showing the green fluorescence indicative of an EGFP-EV71 infection were assessed as FITC^+^ cells. The gating strategy for each cell type is listed in Supplemental Table 2 and Supplemental Figure 1. To identify the CD141^+^ DC subset, the cells were stained with APC-conjugated anti-CD141 (Biolegend, San Diego, CA, USA), PE-conjugated anti-CD11c (Biolegend), and PerCP-Cy5.5-conjugated anti-HLA-DR (BD Biosciences). To identify the CD11b^+^ DC subset, the cells were stained with APC-conjugated anti-CD11b (Biolegend), PE-conjugated anti-CD11c, and PerCP-Cy5.5-conjugated anti-HLA-DR. To identify the plasmacytoid DCs (pDC), the cells were stained with APC-conjugated anti-CD123 (BD Biosciences), PE-conjugated anti-CD11c, and PerCP-Cy5.5-conjugated anti-HLA-DR. To identify monocyte-derived DCs (moDC), the cells were stained with PE/Cy7-conjugated anti-CD14 (Biolegend), PE-conjugated anti-CD11c, and APC-conjugated anti-CD11b. To identify macrophages, the cells were stained with PE/Cy7-conjugated anti-CD14, PE-conjugated anti-CD163 (BD Biosciences), and APC-conjugated anti-CD11b. To identify mature DCs, the cells were stained with PE-conjugated anti-CD83 (BD Biosciences). Isotype-matched IgGs were used concurrently as isotype controls and negative controls lacking antibodies were also included.

### EV71 virus infection in macaques passively immunized with a specific antibody against EV71

Of the nine neonatal rhesus macaques, the positive control group of three were challenged directly with EV71 (FY-23 strain, 10^4.5^ CCID_50_/macaque), while the three in the experiment group were challenged with EV71 1 h after they were injected intravenously with the anti-EV71 antibody (1,500 IU/macaque) using the purified antibody product originating from macaques previously immunized with the EV71 inactivated vaccine. The amount of antibody used was based on an average body weight of about 800 g for the individual macaques, thereby indicating a blood volume of 70–80 ml *in vivo*, and an antibody concentration of 18–20 IU distributed in 1 ml. Here, one IU of serum is defined as a 1:8 dilution against 200 CCID_50_ in a 100 μl volume; consequently, 1,500 IU should lead to a final antibody titer of 1: 16/100 μl. The three negative control group macaques were injected with an anti-EV71 antibody (1,500 IU/macaque) intravenously without viral challenge. Blood samples from the nine macaques were collected daily. Viral loads in plasma, blood cells, and whole blood from the macaques were measured. Sera were isolated for measurement of the neutralizing antibody titers.

### ELISPOT assay for EV71-specific IFN-γ secreting cells

The Monkey IFN-γ ELISPOT Kit (Cytech, Hong Kong, China) was employed as per the manufacturer's protocol to assay the IFN-γ-secreting cells. Briefly, a 96-well polyvinylidene difluoride-backed plate was pre-coated with an anti-IFN-γ mAb and incubated overnight at 4°C. The plate was blocked for 1 h at 37°C. PBMCs were dispensed at a predetermined density in duplicate wells. A total of 10 μg/ml peptide solution was added to stimulate the effector cells. The peptide used to assay the virus-specific IFN-γ-secreting cells was designed using PREDEPP (http://margalit.huji.ac.il/Teppred/mhc-bind) and PROPRED (http://imtech.res.in/raghava/propred/) epitope prediction software. Next, the ASSNASDESMI peptide from the VP1 domain was selected and certified as an effective stimulator. The peptides were determined to be ≥85% pure by HPLC and mass spectrometry analyses. The plate was then incubated at 37°C for another 24 h. After incubation, the cells were removed, and the colors were developed according to the manufacturer's protocol. The colored spots were counted with an automated ELISPOT reader (CTL, Cleveland, OH, USA). The spot-forming cells represent EV71 epitope-specific IFN-γ-secreting cells.

### Statistical analyses

Statistical significance was determined using two-tailed unpaired Student's *t*-tests and GraphPad Prism 5 software (GraphPad Software, San Diego, CA, USA). The data were considered significant when *p* < 0.05.

## Results

### Construction of a fluorescently-labeled EV71 chimera and evaluation of its biological properties

A previous report indicated that biological properties of fluorescently-labeled EV71 chimera were indistinguishable from the parental virus in their plaque morphology and growth kinetics, and the chimera was useful for studying viral pathogenesis (Shang et al., 2013). Thus, based on a primary analysis of EV71 genes, we aimed to insert the EGFP gene in front of the VP4-encoding gene to construct EGFP-EV71 through introducing a gene fragment-encoding peptide (AITTLGS) capable of being recognized as a cleavage site by the viral-encoded 2A enzyme (Figure 1A). The chimera cDNA with the EGFP gene fragment was then cloned downstream of the sp6 start codon for RNA transcription in the sPS64 vector. The transcribed RNA was subsequently transfected into Vero cells. Typical CPEs were observed on day 3 after transfection, a result consistent with the timing of green fluorescence expression in these cells (Supplemental Figure 2).

Biological analyses of the live virus that resulted from transfection with the EGFP-EV71 genome did not reveal any prominent differences compared with the WT-EV71 virus in terms of the dynamic profile of viral growth, the level of neutralization by an anti-EV71-specific antibody, or the plaque morphology phenotype in Vero cells (Figures 1B–D). Additionally, EGFP was clearly expressed during the proliferation of EGFP-EV71 in Vero cells (Supplemental Figure 2). The results of further experiments, in which neonatal rhesus macaques were challenged with the virus via the respiratory tract (spray application to the nasal cavity) or the alimentary tract (in drinking water), indicate that marked vesicles were observed in animals challenged by either WT-EV71 or EGFP-EV71 viruses via the respiratory tract, but not in the animals challenged by either of these viruses via the alimentary tract, which is in agreement with the findings of a previous study (Supplemental Table 3) (Zhang et al., 2011). The histopathological observations on tissues from these animals identified injury to the respiratory tract epithelium, as characterized by tracheal cilia breakage, inflamed cell aggregation in the mucosa, blood vessel congestion, lung structure damage, and lung epithelium injury in the animals infected via the respiratory tract, none of which was evident in the animals infected via the alimentary tract. No epithelial injuries were observed in the tonsils, except inflammatory cell infiltration under the tonsil crypt epithelium and in the associated lymph nodes (Supplemental Figure 3). Additionally, the esophageal and intestinal mucosa in the animals infected through either the respiratory or the alimentary tract was not damaged by the infection (Supplemental Figure 3). All of the data from these studies, combined with our previous findings on EV71 infection in neonatal rhesus macaques (Zhang et al., 2011), confirm that this EGFP-labeled EV71 strain shows similar biological properties to WT-EV71.

### Infection of EV71 in respiratory tract epithelium

**Figure 2:**
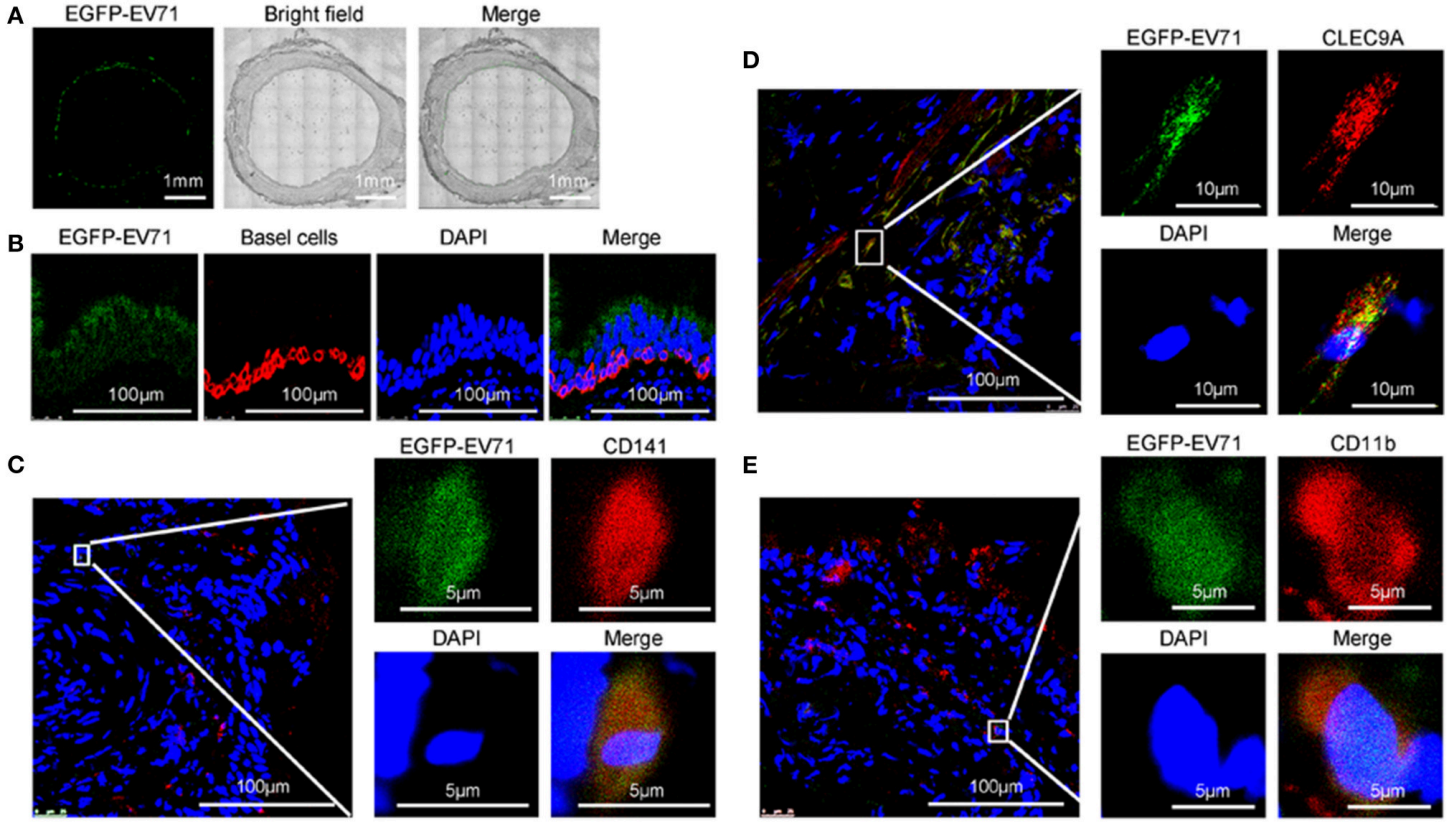
**Assessment of EGFP-EV71 infection in respiratory tract epithelium and DCs. (A)** Images of the trachea from infected animals. Images showing the fluorescence detection of EGFP-EV71 (green). Each image is a mosaic map with 63 spliced images (Magnification, 200×). **(B–E)** Images of the respiratory epithelia from infected animals. Basal cells (red) were detected with an anti-cytokeratin 14 antibody, **(B)** CD141^+^ DCs (red) were detected with an anti-CD141 antibody **(C)**, or an anti-CLEC9A antibody **(D)**, and CD11b^+^ DCs (red) were detected with an anti-CD11b^+^ antibody **(E)**. The tissue samples were obtained from rhesus macaques challenged via the respiratory tract at 3 dpi. Nuclei were stained with DAPI.

Our examination of the tracheas, lungs and the associated lymphoid tissues of the animals challenged with WT-EV71 or EGFP-EV71 via the respiratory tract found elevated viral loads associated with slight injuries (Supplemental Figures 3, 4), and also showed the inflammatory reactions induced by viral replication (Supplemental Figures 3, 4), as has been noticed in our previous studies (Liu et al., 2011; Zhang et al., 2011). Furthermore, by observing the EGFP fluorescence from EGFP-EV71 via confocal microscopy, the tracheal and bronchial epithelial tissues were determined to be the first locations for EGFP-EV71 proliferation during the early phase of EV71 infection (Figure 2A). Specifically, most of the fluorescence signals were localized in the ciliated epithelium of the mucosal surface and in the basal cells of the respiratory epithelium (Figure 2B). Notably, no obvious fluorescence signals from EGFP-EV71 were identified in the esophageal or intestinal mucosal sections.

As shown by confocal microscopy of the areas close to the basal cells, the green fluorescence signals from EGFP-EV71 appeared to co-localize with the red fluorescence emitted by fluorescently-labeled anti-CD141 antibodies in the same respiratory epithelial tissue (Figure 2C). CD141 is generally recognized as a cell marker for a subset of human pre-conventional DCs (cDCs) (Rossi and Young, 2005; Haniffa et al., 2013; Dalod et al., 2014). This subset also expresses CLEC9A, which was similarly observed to co-localize with EGFP-EV71 in our experiments (Figure 2D). Additionally, some of the green fluorescence from EGFP-EV71 co-localized with the red fluorescence in the CD11b^+^ cells that were labeled using a fluorescence-labeled anti-CD11b antibody (Figure 2E). Collectively, these findings suggest that EGFP-EV71 infects the epithelium of the respiratory mucosa and subsequently enters the pre-cDC CD141^+^ and CD11b^+^ subsets in the mucosa through an active or passive pathway. This possibility is consistent with the findings of previous reports describing the ability of EV71 to infect DCs (Lin et al., 2009; Wang et al., 2013). We also traced the fluorescence signal in other cells of the tracheal and bronchial tissues, where our observations were especially focused on macrophages because of the roles they play in phagocytosis and on antigen presentation cells similar to DCs. However, few macrophages emitted a fluorescent signal, and no macrophages involved in the EV71 infections were identified, while few EGFP-EV71 proliferation was observed in B and T cells (**Figure 5**).

### Dynamic distribution of EV71 in the lymphoid tissues around the respiratory tract

**Figure 3:**
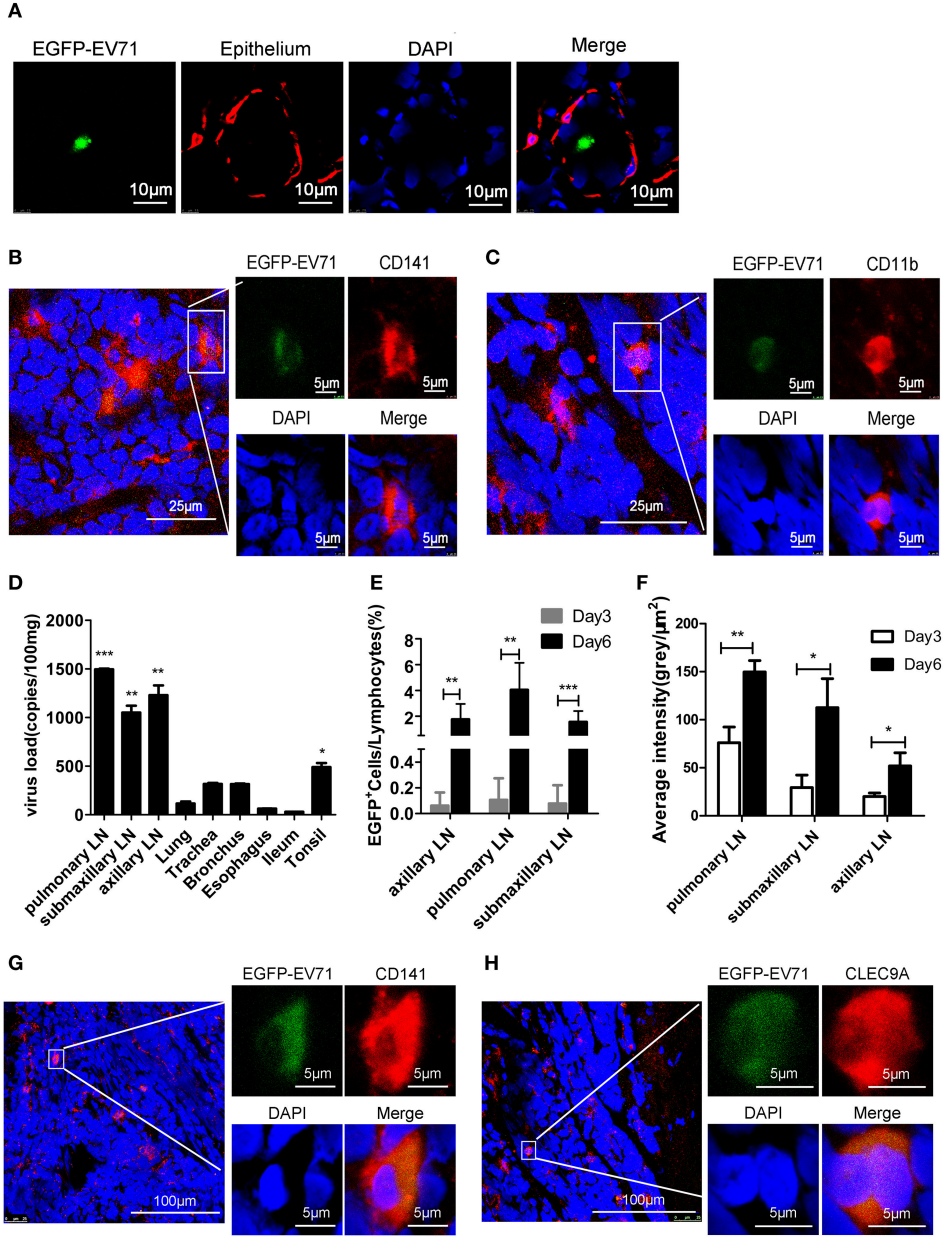
**Assessment of EGFP-EV71 proliferation in tonsils and lymph nodes. (A–C)** Images of the tonsils from infected animals. Epithelium tissue (red) was detected with an anti-cytokeratin 14 antibody **(A)**, CD141^+^ DCs (red) were detected with an anti-CD141 antibody **(B)**, and CD11b^+^ DCs (red) were detected with an anti-CD11b^+^ antibody **(C). (D)** Viral loads in the lymph nodes, tonsils and other organs from the infected animals at 3 dpi, which is during the early stage of infection. The data are presented as the mean ± SD, *n* = 3; *t*-test, two-tailed; ^*^p < 0.05, ^**^p < 0.01, ^***^p < 0.001. **(E)** Results from a flow cytometry analysis of the percentage of EGFP-EV71 infected cells in the lymph nodes on days 3 and 6 pi. The data are presented as the mean ± SD, *n* = 3; *t*-test, two-tailed; ^**^p < 0.01, ^***^p < 0.001. **(F)** Viral loads in the lymph nodes on days 3 and 6 pi. The data are presented as the mean ± SD, *n* = 3; *t*-test, two-tailed; ^*^p < 0.05, ^**^p < 0.01. **(G,H)** Images of the lymph nodes from infected animals. Representative confocal fluorescence images of EGFP-EV71 expression (green) in CD141^+^ DCs (red) labeled with an anti-CD141 antibody **(G)** or an anti-CLEC9A antibody **(H)**. The tissues samples were obtained from rhesus macaques challenged via the respiratory tract at 3 dpi.

Because EGFP-EV71 was observed in tracheal epithelium DCs, we next investigated the dynamic distribution of EV71 in the lymphoid tissue around the respiratory tract. First, based on the reported data suggesting the tonsillar crypt as a portal for viral entry (He et al., 2014), the observation from confocal microscopy confirmed the green fluorescence signal from EGFP-EV71 in tonsillar tissue (Figure 3A). Furthermore, this fluorescence signal co-localized with the red fluorescence signal emanating from antibodies against CD11b and CD141 (Figures 3B,C). These results suggest that the virus does exist in tonsil tissue, which is an important lymphoid tissue for the respiratory and alimentary tracts. Therefore, we hypothesized that EV71 was transferred *in vivo* through lymphokinesis. To investigate this further, the viral load in tonsils and various lymph nodes around the respiratory tract was measured using quantitative reverse-transcription PCR (qRT-PCR) with gene-specific VP1 primers. The results showed that there were much higher viral loads in the lymph nodes and tonsils than in the other organs on day 3 post-infection (pi) (Figure 3D), suggesting that, early in infection, these lymphoid tissues are an important site for viral proliferation (Figures 3E,F). Additionally, in the pulmonary lymph nodes, which had a high viral load, the confocal microscopy observations indicated that the virus still proliferated primarily in the CD141^+^DC subset (Figures 3G,H). Only minimal EGFP fluorescence signals from EGFP-EV71 were captured from the other cell types. These findings imply that EV71 has a preference for these cells in lymphoid tissues.

### Characterization of viremia in macaques infected with EV71

**Figure 4:**
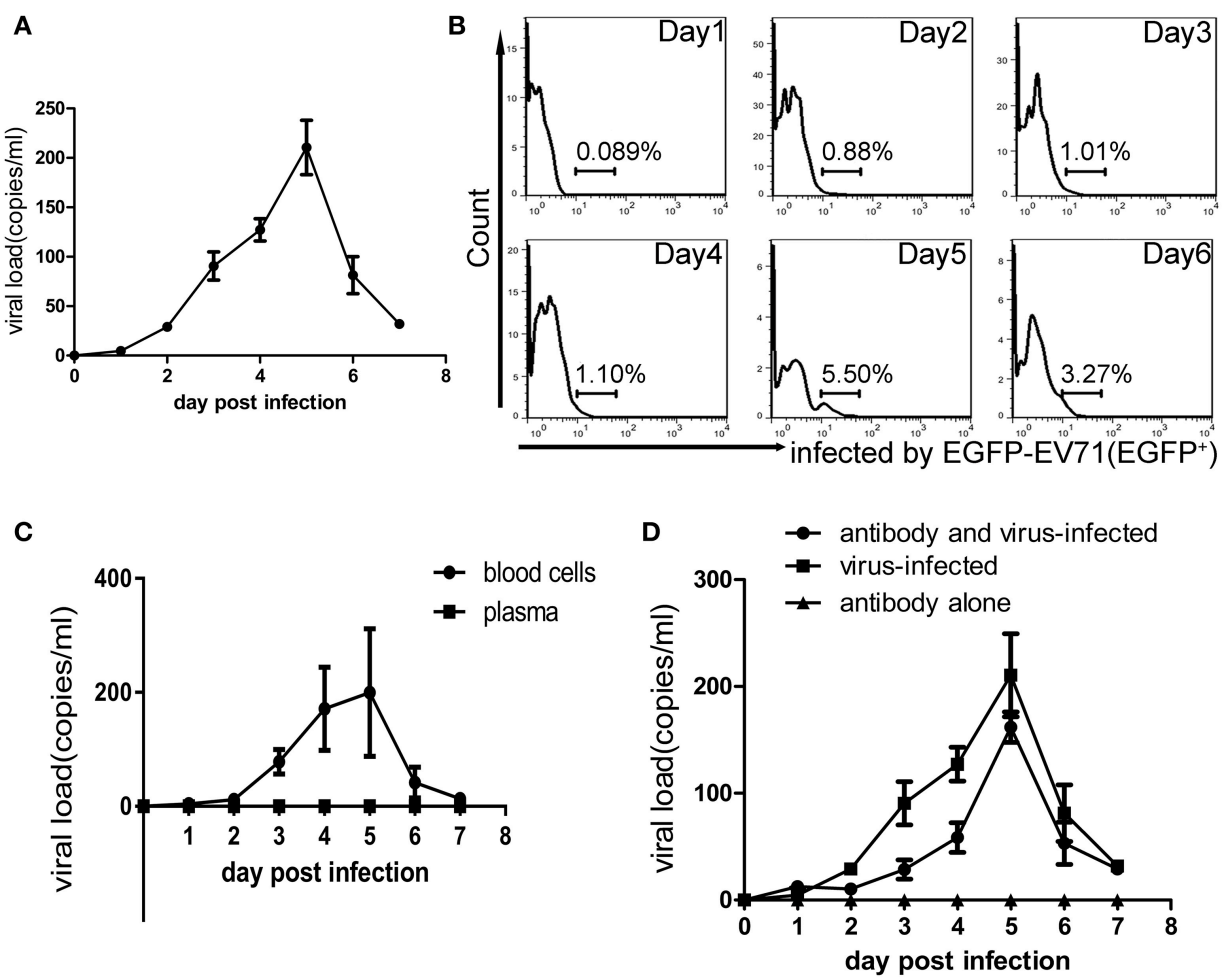
**Viremia of macaques infected with EV71. (A)** Virus loads in the blood obtained from infected macaques within 7 dpi. **(B)** The presence of EGFP-EV71 in PBMCs (as assessed by the expression of EGFP) from infected macaques during days 1–6 pi was analyzed using flow cytometry, and the representative dot plots are shown. **(C)** Viral loads in the blood cells and plasma from infected macaques within 7 dpi. **(D)** Viral loads in whole blood from the virus-infected group and in animals infected with the virus after passive immunization within 7 dpi. The data presented are the mean ± SD of the independent experiments from three animal, *n* = 3.

In the infectious process of enteroviruses, viremia is usually thought to be a clinical pathological indicator, meaning that the virus is transferred to target tissues via the blood circulation after viral replication in the primary infection site (Rittichier et al., 2005). In our previous work, viremia was identified in macaques infected by EV71 (Liu et al., 2011; Zhang et al., 2011), and the virus was found to exist in PBMCs (Wang et al., 2013). In the work described here, viremia was identified again by viral load measurements using q-RT-PCR and EGFP^+^ counting of the cells in blood samples and these showed a peak phase on days 3–5 pi (Figures 4A,B). Also, the virus distribution in the blood was determined by analysis of the blood plasma and blood cells, via their centrifugal separation from blood samples collected at various time points. The analysis confirmed that the virus did exist in blood cells during the viremia period but not in the plasma (Figure 4C). Because this result challenges the traditional view that specific neutralizing antibodies in serum can interact with the virus (De Gregorio and Rappuoli, 2012), it needed further evidence to support it. Therefore, we performed a new infection experiment with nine macaques divided into three groups as described in the Materials and Methods Section. Macaques in each experimental group received a specific anti-EV71 antibody, the purified product of which originated from macaques immunized with an inactivated EV71 vaccine. After receiving 1,500 IU of the antibody via intravenous injection, blood samples from each macaque were collected daily and the result of neutralizing antibody titer analysis showed that the macaques had serum antibody levels of 1:8–16, which is a sufficient amount to protect them against viral challenge (see Section Materials and Methods). Compared with the positive control group that received no antibody inoculations, the observations in the experimental group suggested that clinical pathological features such as similar vesicular lesions in the lips, fervescence, and viremia were present in the animals, while the negative controls, which only received antibody, did not show any clinical signs (Table 2; Figure 4D). This experiment provides stronger supportive evidence for the virus existing in the blood cells, as was suggested above.

**Table 2:** **Clinical manifestations observed in neonatal rhesus macaques**.

**Pattern**	**Body temperature (°C)^a^ (day post infection)**							**Vesicles**^b^		
	**1**	**2**	**3**	**4**	**5**	**6**	**7**	**Hand**	**Foot**	**Mouth**
Experimental group (Antibody + virus-infected)	38.6	39.1	39.6^↑^	39.8^↑^	39.9^↑^	39.7^↑^	39.8^↑^	Y	Y	Y
Control group (Virus-infected)	38.5	38.9	39.5^↑^	39.7^↑^	39.7^↑^	39.4^↑^	39.3^↑^	Y	Y	Y
Control group (Antibody injection alone)	38.7	38.9	39.1	38.6	38.9	39.1	38.6	N	N	N

a *The data are presented as the means of independent experiments from three animals*.

b *N, not observed Y, observed*.

↑ *rise in the body temperature*.

### EV71 prefers the CD141^+^ subset of pre-cDCs

**Figure 5:**
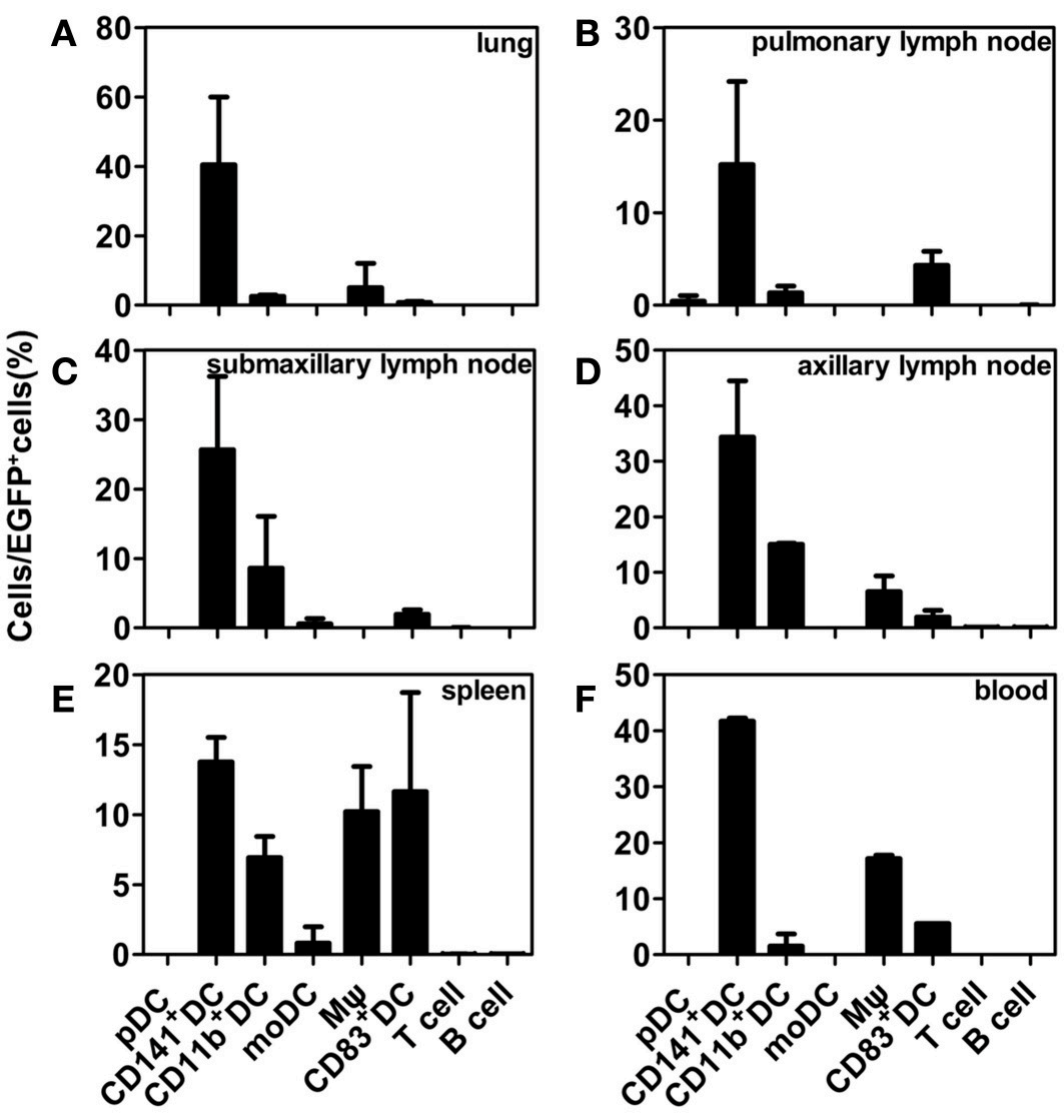
**Flow cytometry analysis of EGFP-EV71-infected DC subsets**. Distribution of EGFP-EV71-infected DC subsets in the lungs **(A)**, pulmonary lymph nodes **(B)**, submaxillary lymph nodes **(C)**, axillary lymph nodes **(D)**, spleen **(E)**, and blood **(F)**. The data are presented as the mean ± SD, *n* = 3. The tissues samples were obtained from rhesus macaques challenged via the respiratory tract at 6 dpi.

DCs, the key regulators of immune activation, develop in the bone marrow from the common DC precursors that give rise to plasmacytoid DCs (pDCs) and to the intermediate cells known as pre-cDCs (Dalod et al., 2014). After observing that EV71 infects DCs in the trachea and respiratory lymph nodes and appears to co-localize with CD141^+^ DCs, we aimed to clarify if this DC subset is preferentially infected by EV71. The results of our flow cytometry experiments confirm that the CD141^+^ pre-cDC subset is the main DC population preferentially infected by EV71 (Figure 5). In the lungs, one of the organs primarily infected by EV71 when infection occurs via the respiratory tract, ~40% of the EGFP-positive cells belonged to the CD141^+^DC subset on day 6 pi (Figure 5A). At this time, the CD141^+^ rates were ~15% of those of the EGFP-positive cells in the pulmonary lymph node and of about 25, 34, 15, and 40% of the EGFP-positive cells in the submaxillary lymph node, axillary lymph node, spleen, and blood, respectively (Figures 5B–F). However, approximately 8.5, 15, and 7% of the EGFP-positive cells from the submaxillary lymph node, axillary lymph node, and spleen, respectively, belonged to the CD11b^+^ DC subset (Figures 5C–E), and about 6.5, 10, and 18% of the EGFP-positive cells from the axillary lymph node, spleen, and blood, respectively, were macrophagocytes (Figures 5D–F). Interestingly, about 15 and 6% of the EFGP-positive cells from the spleen and blood, respectively, were mature DCs (CD83^+^DCs; Figures 5E,F), but few EFGP-positive T and B cells were found.

### EV71 infects DCs cultured *In vitro*

**Figure 6:**
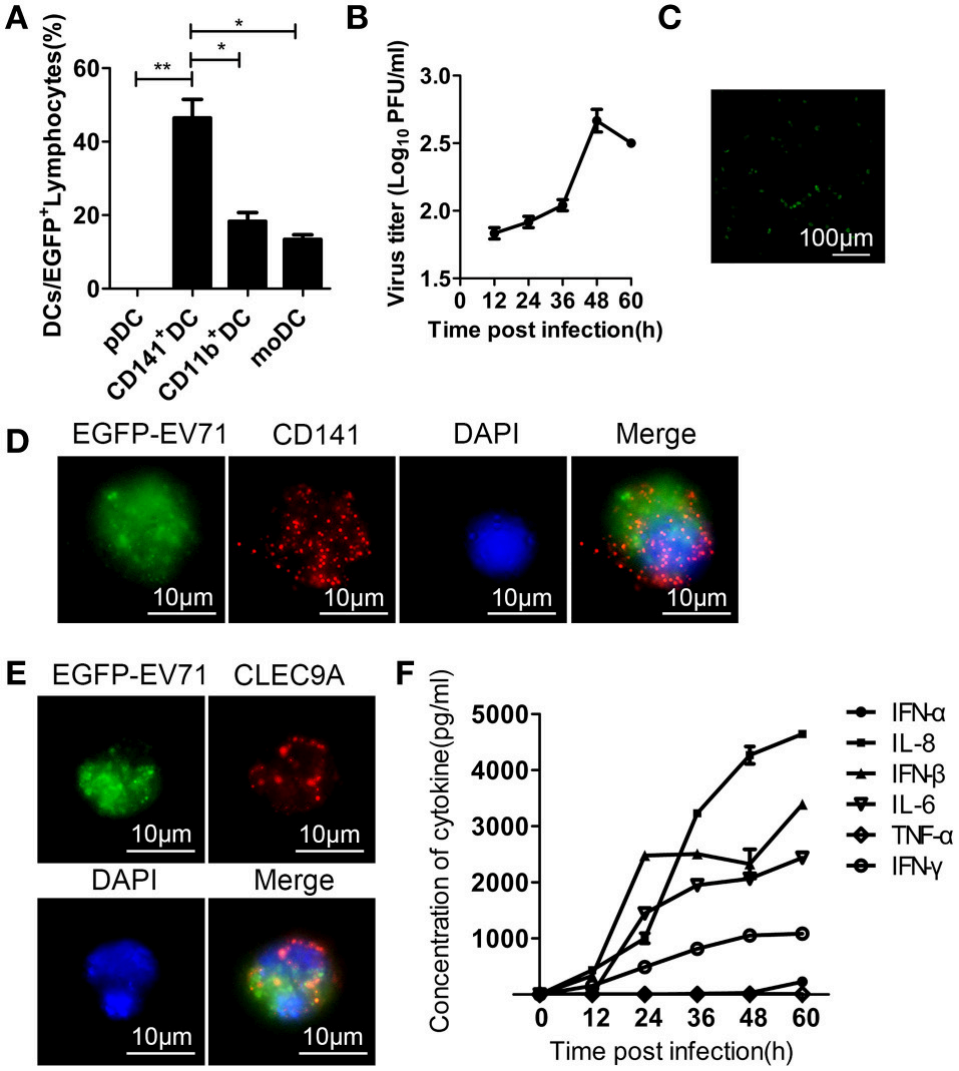
**Assessment of EGFP-EV71 infection of cultured DCs. (A)** Flow cytometry was performed to detect the distribution of DC subsets infected by EGFP-EV71 *in vitro*. The data are presented as the mean ± SD, *n* = 3; *t*-test, two-tailed; ^*^p < 0.05, ^**^p < 0.01. **(B)** Viral titration analysis of EGFP-EV71-infected DCs. The data are presented as the mean ± SD, *n* = 3. **(C)** Cultured DCs were infected by EGFP-EV71, and their green fluorescence expression was assessed by fluorescence microscopy. **(D–E)** *In vitro* EGFP-EV71-infected CD141^+^ DCs (red) were labeled with an anti-CD141 antibody **(D)** and anti-CLEC9A antibody **(E)**. **(F)** Cytokines detected in the supernatant of EGFP-EV71-infected cultured DCs.

The distribution of EGFP-EV71 in several subsets of the DC population suggests that the virus has a specific preference for DCs when entering the animal host before infecting other tissues. We next used the cultured DC subsets isolated by flow cytometry to investigate viral infection in these cells and to examine the influence of this infection on their biological properties. The results confirmed an EV71 preference for the CD141^+^ subset of pre-cDCs, whereas the other DC subsets had lower rates of viral infection based on the fluorescent signal detected from EGFP-EV71 and the viral titration (Figures 6A,B). Green fluorescence was observed in cultured DCs at 48 h pi (Figure 6C), and the presence of green fluorescence in CD141^+^ DCs indicates that these cells were infected by EGFP-EV71 (Figures 6D,E). Additionally, various cytokines were detected in the supernatants from these infected DCs, indicating that the levels of some of the cytokines associated with immunoregulation increased after EGFP-EV71 infection (Figure 6F). Notably, our conclusions from these experiments are consistent with our previous finding that EV71 infection does not impact antigen presentation and subsequent T cell activation by DCs (Lin et al., 2009; Wang et al., 2013).

## Discussion

In clinical epidemiology, EV71 is one of the main pathogens responsible for HFMD (Ho et al., 1999; Lee et al., 2009), and this virus can cause illness via extensive infections of either the respiratory or alimentary tract in children (McMinn, 2002; Rajtar et al., 2008). Although the pathogenesis of EV71 is not fully understood (Huang and Shih, 2014), our previous data obtained from neonatal rhesus macaques suggest that the development of viremia results in the infection and pathological process being targeted to particular organs, specifically the central nervous system (CNS) (Dong et al., 2010; Zhang et al., 2011). Thus, studying the processes and associated mechanisms concerning how the virus progresses from a local infection to viremia is essential for understanding EV71 pathology. The EGFP-EV71 virus constructed here effectively expresses EGFP during its replication in cells, which greatly assisted our investigations of EV71 infection in neonatal rhesus macaques. Importantly, this constructed virus shows similar biological characteristics to those of WT-EV71.

By examining the proliferation of EGFP-EV71 during infection via the respiratory and alimentary tracts in neonatal macaques, we found that the virus tends to proliferate not only in the ciliated epithelium and basal cells of the epithelial tissue in the respiratory tract and subsequently damages the epithelial tissue, but also in the lymphoid tissues around the respiratory tract. The study reported by He and colleagues suggested that the tonsillar crypt epithelium was an important extra CNS site for viral replication in EV71 encephalomyelitis, and their study showed the damage caused by the epithelial tissue-associated inflammatory reaction and the high viral load in the tonsils (He et al., 2014). Our results showed a similar increasing viral load and inflammatory cell infiltration in the tonsillar tissue, except for the damage we observed in the epithelium, and also identified the existence of the virus in the CD141^+^ cells of the tonsils by confocal microscopy, the same as in the other lymph nodes around the respiratory tract. These observations offer the possibility that EV71 infection might be initiated in the respiratory tract and its associated lymphoid tissues. In contrast, EGFP-EV71 had very low levels of proliferation in the epithelium of the alimentary tract. Although, EV71 is classified as an enteric pathogen (Hsiung and Wang, 2000; Pallansch and Ross, 2001), our study could not determine if the failure to induce infection via the alimentary tract was the result of characteristics specific to the macaque species used in the experiments. However, our observations of the tracheal tissue and its associated lymphoid tissues from the infected animals suggest that the pre-cDC population, mainly the CD141^+^ subset of it, acts as a relay system for transferring the virus to its next target. Therefore, proliferation of EV71 in the tracheal mucosal epithelium and subsequent cell damage might stimulate an innate immune response through the interaction of viral pathogen-associated molecular patterns with their corresponding pattern recognition receptors in the epithelial cells and give rise to the recruitment of DCs (Schon-Hegrad et al., 1991; Pathinayake et al., 2015). However, based on this possibility, macrophages in the respiratory tract tissues should be recruited and undertake phagocytosis (Blank et al., 2017). No captured fluorescence signal from these cells seemingly suggests they play a minor function in the early phase of the viral infection. But identifying the virus in the cells of the lymph nodes, spleen, and blood indicated their involvement in the immune response against the virus.

Although, it is not clear how the virus enters the CD141^+^ DC population, our data indicate that this DC population may act not only as a carrier for transferring the virus to various organs through the lymph nodes and peripheral blood, but also as an important site of virus proliferation during early infection. This highlights the route and manner of EV71 infection as well as the development of viremia through virus proliferation in this DC subset during the early stage of infection. Importantly, this challenges the concept of neutralizing antibodies in serum being capable of blocking virus transfer thereby leading to termination of the viral infection, which was thought to be the mechanism of action of the viral vaccine (De Gregorio and Rappuoli, 2012; Furman and Davis, 2015). However, the data from our previous study on inactivated EV71 confirmed that the viral infection did not induce viremia, which was detected in the q-RT-PCR assay for viral genomic RNA in the macaques immunized with the vaccine (Zhang et al., 2015); this indicates that, combined with the observation that the virus exists in DCs, the viral infection would had been controlled before the virus entered the blood circulation. Consequently, the finding described here does appear to be reconcilable with the concept of antibodies capable of neutralizing viruses in the blood circulation.

We aimed to determine whether EV71 infection in the CD141^+^ DC subset is involved in the pathogenesis of viral infection, and we also investigated whether the viral infection in this DC population interferes with the development of an independent immune response against EV71. Clinical manifestations of EV71 infection include increased expression levels of some inflammatory cytokines, such as IL-6 and TNF-α, which are also induced by LPS stimulation. The results of our experiments using CD141^+^ DCs isolated from macaques and cultured *in vitro* indicate that the EV71-infected cells have the same cytokine expression profile as the positive control cells that were stimulated with LPS. Additionally, our findings that EGFP-EV71-infected rhesus macaques generate both EV71-neutralizing antibodies and that T cells produce IFN-γ in an ELISPOT assay against an EV71-specific antigen peptide (Supplemental Figure 5) confirm that infection with this virus induces an immune response. Our results support the following conclusions: (1) EV71 prefers to proliferate in the CD141^+^ and CD11b^+^ subsets of the pre-cDC population and to use these cells for its transmission during the early stages of infection in rhesus macaques and, (2) the proliferation and transmission of EV71 in these specific DC subsets during the infective process is not involved in the pathogenesis of the viral infection, nor does it interfere with the development of an independent immune response against EV71. Further studies are warranted to determine the details of the progression of EV71 infection in DCs and the subsequent pathological results and immune response to this infection.

## Author contributions

QL, TZ, ZZ designed the research; TZ, ZZ, YZ, MF, SF, LW, XW, and QW performed the research; LL, XZ, JW, YL, and ZH contributed new reagents/analytic tools; TZ, YZ, SL, and HY analyzed the data; TZ and QL wrote the paper.

## Funding

This work was supported by the National Basic Research Program (2011CB504903), the State Project for Essential Drug Research and Development (2012ZX09101319 and 2014ZX09102042), the Technology Development Research Institutes (2013EG150137), the National Natural Science Foundation (81171573 and 31370192), and the Yunnan Province Project (2013FA024, 2012ZA009 and 2014FB191).

### Conflict of interest statement

The authors declare that the research was conducted in the absence of any commercial or financial relationships that could be construed as a potential conflict of interest.
