# Supplementary material for: Dynamic Interaction of Enterovirus 71 and Dendritic Cells in Infected Neonatal Rhesus Macaques
Source: Front Cell Infect Microbiol. 2017 May 10;7:171. doi: 10.3389/fcimb.2017.00171 (PMC5423916; doi:10.3389/fcimb.2017.00171)
Supplement: Supplementary file 1 [file DataSheet1.PDF]

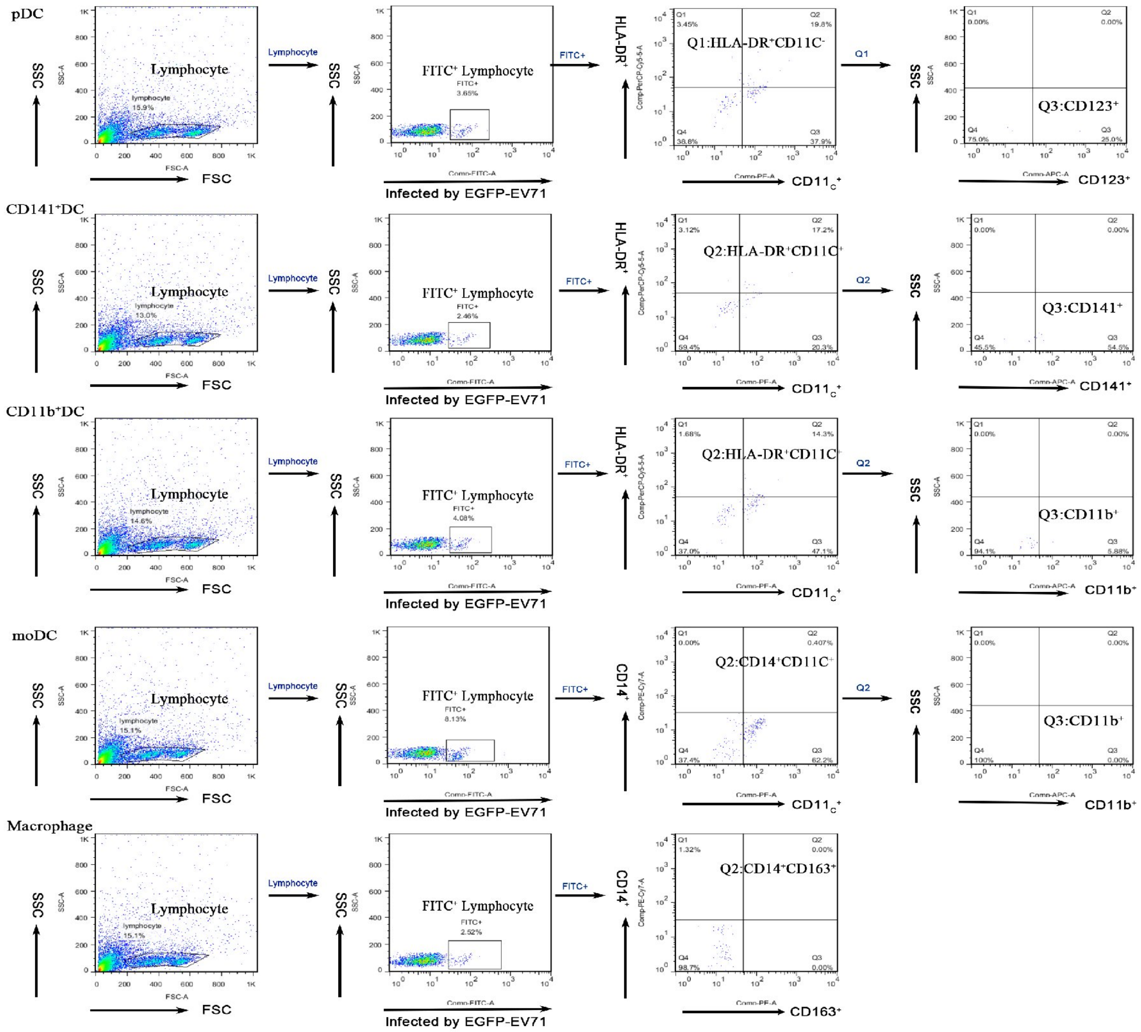

Supplemental Fig 1. The gating strategy for each cell type

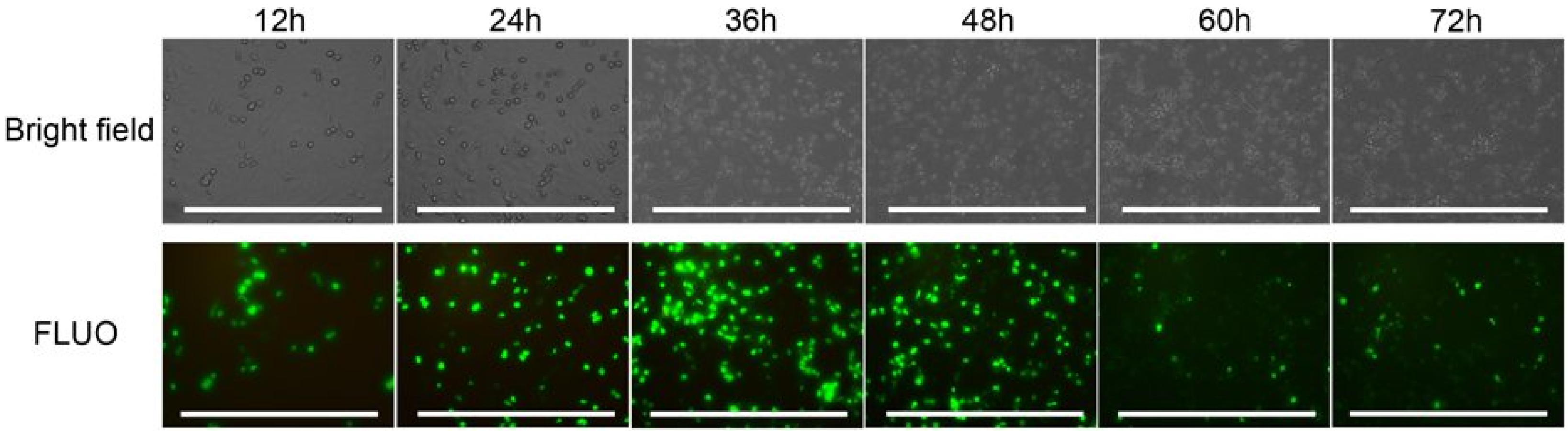

Supplemental Fig 2. CPE observations. Representative images of CPE (bright field) and fluorescence (FLUO) observations in Vero cells at different hours post-infection by EGFP-EV71 (MOI = 1), Bar=500μm.

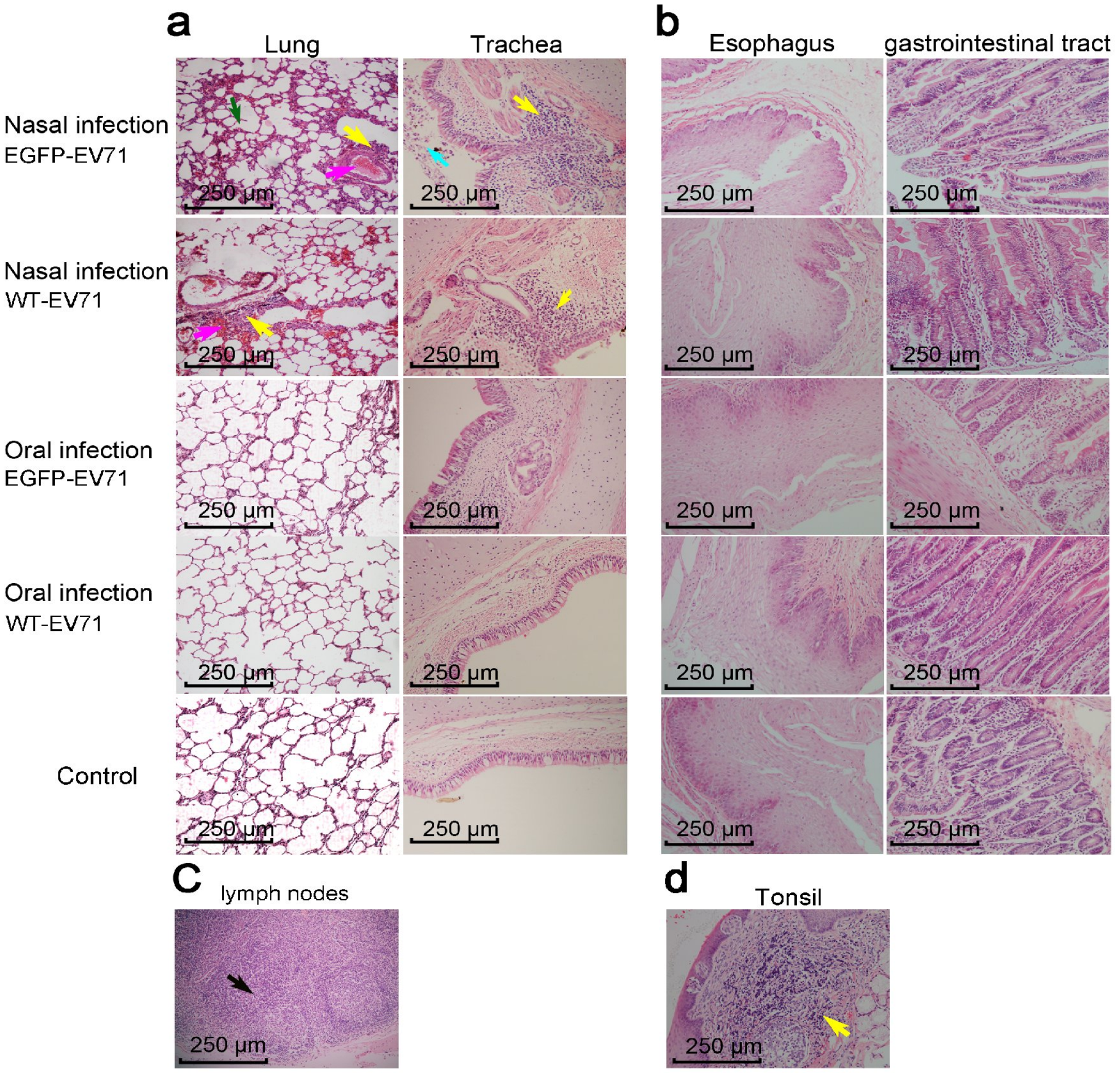

Supplemental Fig 3. Pathologic manifestations observed in EV71-infected neonatal rhesus macaques. (a) Images of the histopathologic characteristics of the respiratory tract. The tissues samples were obtained from rhesus macaques challenged at 142 h post-infection. **(b)** Images of the histopathologic characteristics of the alimentary tract. The tissues samples were obtained from rhesus macaques challenged at 142 h post-infection. **(c)** Images of the histopathologic characteristics of lymph nodes around respiratory tract. The lymph nodes were obtained from rhesus macaques challenged via the respiratory tract

at 142 h post-infection. **(d)** Images of the histopathologic characteristics of tonsil. The tissues samples were obtained from rhesus macaques challenged via the respiratory tract at 142 h post-infection. Blue arrow, cilia desquamation; yellow arrow, inflammatory cell aggregation; purple arrow, blood vessel congestion; green arrow, lung structural damage; and black arrow, expansion of germinal center.

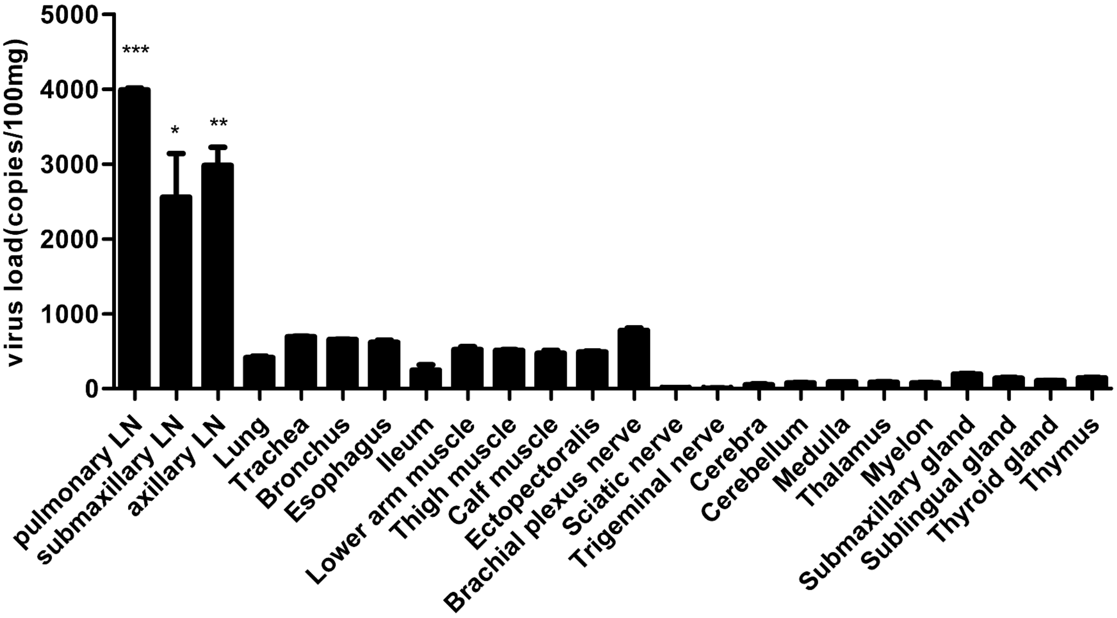

Supplemental Fig 4. Viral loads in the lymph nodes and other organs during the early stage of EV71 infection. The viral loads in the pulmonary lymph node, submaxillary lymph node, axillary lymph node, and other organs on day 3 p.i. The data are presented as the mean ± SD, n=3; *p < 0.05, **p < 0.01, ***p < 0.001.

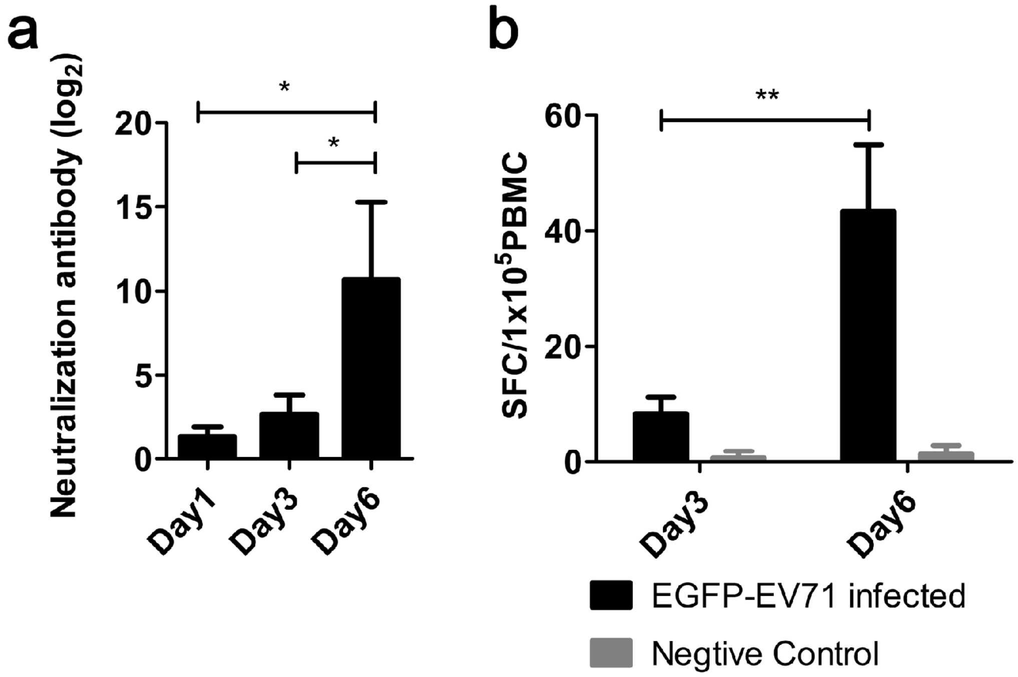

Supplemental Fig 5. Assessment for neutralizing antibody and IFNγ-secreting cells of EGFP-EV71-infected rhesus macaque. (a) Results from a neutralizing antibody test of EGFP-EV71-infected rhesus macaque peripheral blood samples. **(b)** Amount of IFNγ-secreting cells in the peripheral blood of EGFP-EV71-infected rhesus macaques as assessed by an ELISpot assay. The data are presented as the mean ± SD, n=3; *p < 0.05, **p < 0.01.

Supplemental Table 1. Antibodies used in the Immuno-fluorescence studies

Labeled cells	First antibody	Second, fluorescence-labeled antibody
$CD141^+$ cells	Anti-CD141 antibody (Abcam, Cambridge, UK)	Alexa Fluor®594 anti-rabbit (Life Technologies, Carlsbad, CA, USA)
$CLEC9A^+$ cells	Anti-CLEC9A antibody (Abcam, Cambridge, UK)	Alexa Fluor®594 anti-mouse (Life Technologies, Carlsbad, CA, USA)
$CD11b^+$ cells	Anti-CD11b antibody (Biolegend, San Diego, CA, USA)	Alexa Fluor®594 anti-mouse (Life Technologies, Carlsbad, CA, USA)
Basal cells	Anti-Cytokeratin14 antibody (Abcam, Cambridge, UK)	Alexa Fluor®594 anti-rabbit (Life Technologies, Carlsbad, CA, USA)

Supplemental Table 2. FACS staining

Subsets	Anti-body	+/-
pDC	APC-CD123	+
	PE-CD11c	-
	PerCP-Cy5.5—HLA-DR	+
141^{+}DC	APC-CD141	+
	PE-CD11c	+
	PerCP-Cy5.5—HLA-DR	+
$11b^{+}$DC	APC-CD11b	+
	PE-CD11c	+
	PerCP-Cy5.5—HLA-DR	+
moDC	PE/Cy7-CD14	+
	PE-CD11c	+
	APC-CD11b	+
macrophage	PE/Cy7-CD14	+
	PE-CD163	+
	APC-CD11b	+

Supplemental Table 3. Clinical manifestations observed in neonatal rhesus macaques after infection with EGFP-EV71 or wild type EV71

Pattern[a]	Body temperature (°C) (day post infection)						Vesicles[b]			Virus loads in blood (day post infection) (unit:copies/ml)					
	1	2	3	4	5	6	Hand	Foot	Mouth	1	2	3	4	5	6
Nasal infection (E) [c]	37.0	38.0	38.5	39.9	39.8	39.8	Y	Y	Y	0	12	33	175	300	180
Oral infection (E) [c]	37.2	38	38.3	38.5	38.5	38.9	N	N	N	3	15	32	51	52	46
Nasal infection (w)	37	37.5	38.9	38.7	39	39.7	Y	Y	Y	5	14	15	92	343	150
Oral infection (w)	37.1	37.3	38	37.8	37.8	37.6	N	N	N	3	18	21	43	65	14

[a] (E): EGFP-EV71; (W): wild-type EV71;

[b] N: not observed; Y: observed;

[c] The two group data are presented as the means of independent experiments from three animals, n=3;
